# Assessment of Cancer Care Costs in Disease-Specific Cancer Care Pathways

**DOI:** 10.3390/ijerph17134765

**Published:** 2020-07-02

**Authors:** Mattia Altini, Laura Solinas, Lauro Bucchi, Nicola Gentili, Davide Gallegati, William Balzi, Fabio Falcini, Ilaria Massa

**Affiliations:** 1 Healthcare Administration, Istituto Scientifico Romagnolo per lo Studio e la Cura dei Tumori (IRST) IRCCS, 47014 Meldola, Italy; mattia.altini@irst.emr.it (M.A.); william.balzi@irst.emr.it (W.B.); 2 Management and Accounting Unit, Istituto Scientifico Romagnolo per lo Studio e la Cura dei Tumori (IRST) IRCCS, 47014 Meldola, Italy; sol.lau85@gmail.com (L.S.); davide.gallegati@irst.emr.it (D.G.); 3 Romagna Cancer Registry, Istituto Scientifico Romagnolo per lo Studio e la Cura dei Tumori (IRST) IRCCS, 47014 Meldola, Italy; fabio.falcini@irst.emr.it; 4 Information Technology Unit, Istituto Scientifico Romagnolo per lo Studio e la Cura dei Tumori (IRST) IRCCS, 47014 Meldola, Italy; nicola.gentili@irst.emr.it; 5 Cancer Prevention Unit, Morgagni-Pierantoni Hospital, 47121 Forlì, Italy; 6 Unit of Biostatistic and Clinical Trials, Istituto Scientifico Romagnolo per lo Studio e la Cura dei Tumori (IRST) IRCCS, 47014 Meldola, Italy; ilaria.massa@irst.emr.it

**Keywords:** healthcare cost, cancer care cost, care pathway, administrative data

## Abstract

In view of an efficient use of the Italian National Health Service-funded healthcare resources, a novel data-processing strategy combining information from multiple sources was developed in a regional cancer network of northern Italy. The goal was to calculate the annual overall cost of care pathways of six disease groups in 10,486 patients. The evaluation was conceived as a population-based cost description from the perspective of the Italian National Health Service. Costs occurred during a defined time period for a cross-section of patients at varying stages of their disease were measured. The total cancer care cost was €81,170,121 (11.1% of total local health expenditure), with a cost per patient of €7741.17 and a cost per capita of €204.62. Surgical, inpatient and day-hospital medical admissions, radiotherapy, drugs, outpatient care, emergency admissions, and home and hospice care accounted for 21.2%, 24.1%, 6.2%, 28.2%, 14.0%, 0.9%, and 5.4% of the total cost, respectively. The highest cost items included drugs (cost per capita, €22.95; 11.2% of total cost) and medical admissions (€14.51; 7.1%) for blood cancer, and surgical (€14.56; 7.1%) and medical admissions (€13.60; 6.6%) for gastrointestinal cancer. The information extracted allows multidisciplinary cancer care teams to be more aware of the costs of their clinical decisions.

## 1. Introduction

The scientific advances in cancer research of the second half of the 1990s have led to the development of new tools for the diagnosis and treatment of the disease. Screening activities have become widespread. Progress has been made in diagnostic technologies, genetic profiling, and treatments, with the introduction of targeted drugs, the diffusion of multidisciplinary care models, and a growing concentration of treatment in specialist centers [1,2].

The burden of cancer, however, is increasing worldwide [3,4]. In 2015, there have been 17.5 million cancer cases and 8.7 million deaths [3]. Between 2005 and 2015, the number of cases has increased by 33%, with population aging contributing to 16%, population growth to 13%, and changes in age-specific risk to 4% [3].

These two trends have been paralleled by a documented increase in cancer-related healthcare cost [5], although there have been differences between and within countries. In Europe, health expenditure on cancer increased continuously from €35.7 billion in 1995 to €83.2 billion in 2014, and spending on cancer drugs from €7.6 billion in 2005 to €19.1 billion in 2014 [6]. This represents an increasing threat to the sustainability of the national health systems. In the next few years, according to forecasts, this trend will cause further growth in hospital admissions due to cancer [7]. In Europe and elsewhere, these challenges will remain at the top of the agenda of policymakers [7].

The control of risk factors and the diffusion of screening programs [7,8] may have an impact on the incidence of cancer. These efforts, however, are counterbalanced by the risk increase resulting from the population aging [7,9]. The standard strategy for improving cancer care while controlling the costs [10,11,12,13] is to promote the adherence to clinical practice guidelines [14]. A new, and promising, solution is to perform qualitative analyses of medical procedures in order to improve the uptake of research findings into practice [15] and to identify areas of low-value use of resources [16]. A related useful approach is to obtain population-based data on cancer-related health services and their costs in epidemiologically comparable geographic areas, which would enable to identify unwarranted variations caused by non-standard practices.

With this rationale, we have undertaken a research project of which the main goal is to evaluate the costs, the quality of services, and the clinical outcomes of cancer care in a healthcare district of northern Italy. The project, which is part of the ongoing creation of the local cancer care network, is specifically aimed at:

(a) developing indicators of the value of cancer care at the population level, as defined according to the three-fold definition proposed by Gray et al. (allocative value, technical value, personalized value) [17];
(b) developing a strategy for combining health data from multiple sources in order to identify areas of waste of resources;
(c) developing a procedure to calculate the costs along the whole care pathways;
(d) developing indicators for benchmarking initiatives in order to identify unwarranted variations [18]; and
(e) evaluating sustainability issues of cancer care for society as a whole [19]. These objectives require cost descriptions where data are aggregated by disease and care setting [20,21].

The results of a first empirical study, specifically addressing the objective (b), have shown that there is potential room for redistributing resources inappropriately used [16]. This second article focuses on the objective (c).

## 2. Materials and Methods

### 2.1. Setting: The Romagna Cancer Care Network

The Romagna Cancer Institute (Italian: *Istituto Scientifico Romagnolo per lo Studio e la Cura dei Tumori, IRST*) of Meldola is a partnership among three public sector bodies and six private nonprofit organizations [22]. A process of networking between the IRST and the local hospitals and healthcare facilities is underway. At the completion of this design, the IRST will formally act as the hub of a hub-and-spoke system of cancer care delivery. The network will serve a population of about 1,125,000 living in the Romagna healthcare district, which covers three provinces in the Emilia-Romagna Region (northern Italy).

According to the Romagna Cancer Registry, the population of the district has one the highest total cancer prevalence rates in Italy, i.e., 5540 per 100,000 (both sexes combined) versus a national average of 4558 and a range of 2720–6081 [23].

### 2.2. Rationale: The Disease-Specific Cancer Care Workgroups and Pathways

In 2008, in the context of the process of networking, the IRST established six broad disease-specific cancer care workgroups, defined as groups of dedicated physicians from different specialties and different local hospitals who collaborate in real-time as multidisciplinary teams and develop comprehensive, personalized patient plans. The International Classification of Diseases—10th Revision (ICD-10) [24] codes of their target diseases are the following: blood cancer, C81–C85, C88, C90–C96; gastrointestinal cancer, C15–C26, C48; breast cancer, C50; uro-gynecologic cancer, C51–C59, C60–C68; thorax cancer, C33–C34, C37–C39, C45, C78; rare cancers and others, C00–C14, C30–C32, C40, C41, C43, C44, C46, C47, C49, C69–C77, C79, C80, C97.

The workgroups will have to account both for the cost and the outcomes of cancer care. They will have the leading responsibility for the delivery of state-of-the-art care through standardized care pathways and for an efficient use of the budget. The care pathways are defined as general clinical plans aiming at mapping the patient transition through different levels of the local healthcare system [25,26] and at optimizing the use of the Italian National Health Service (INHS)—funded healthcare resources. The workgroups are provided with detailed information on key performance indexes and main cost components, as previously described [16].

### 2.3. Study Objectives

The above background has provided the motivation for this study, of which the objectives were: (a) to develop an extract/transform/load (ETL) procedure, that is, a systematic data retrieval and processing strategy for acquiring cross-sectional information from multiple sources and calculating the amount of all cost components along the care pathways of the six disease groups; and (b) to test this strategy through a pilot study on a population-based series of patients living in one of the three provinces served by the Romagna cancer care network. This work was intended to be a feasibility study, aiming to assess the practical aspects of data retrieval and the overall consistency of the gathered information, and not a conclusive analysis.

### 2.4. Study Design

The study was conceived as a population-based cost description from the perspective of the INHS [20]. It was designed to measure the disease-attributable cost that occurs during a defined time period for a cross-section of patients at varying stages of their clinical history [27].

The study covered the healthcare cost for diagnosis and treatment in a defined series of patients during 2016. The choice of this study year is motivated by the facts that (a) the data for 2016 represent the baseline situation against which to measure subsequent improvements (if any), that are the subject of an ongoing evaluation, and (b) during this 5-year period, no new items of expenditure nor new data sources have been created. Consequently, the ETL tool has not been modified.

Included were all costs borne by any Italian public and private accredited provider for any type of health service. The study was restricted to patients living in the Province of Forlì-Cesena because the local healthcare facilities offer more possibilities of performing direct data checks.

### 2.5. Data Sources

**Figure 1:**
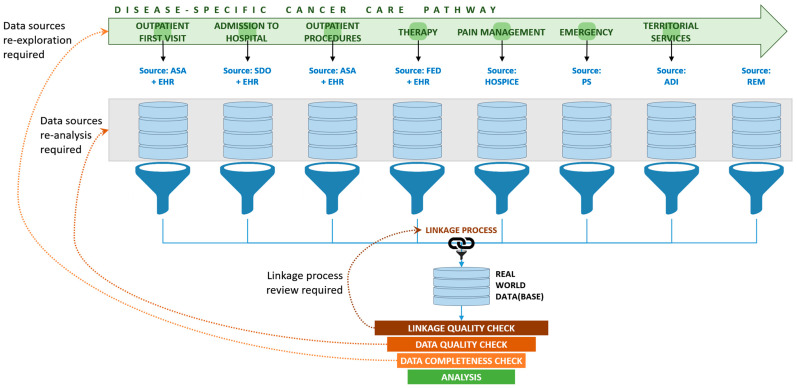
Schematic diagram of the extract/transform/load (ETL) process for acquiring cross-sectional information from multiple sources. Except for the Hospice Database, the data sources are indicated with the standard abbreviation in Italian: ASA indicates Outpatient Specialist Assistance Database; EHR indicates Electronic Health Record, that is, the electronic clinical data record archive at the *Istituto Scientifico Romagnolo per lo Studio e la Cura dei Tumori* (IRST) of Meldola; SDO indicates Hospital Discharge Database; FED indicates Drugs Database; PS indicates Emergency Database; ADI indicates Home Care Database; REM indicates Mortality Registry.

The study was made using nominative individual data with record-linkage processes between multiple databases due to the absence of information on disease diagnosis in many data sources and the absence of a reliable universal identifier of Italian citizens. In Figure 1, the ETL process is schematically depicted. In medicine, ‘real world data’ (bottom part of the diagram) is a technical term indicating data derived from a number of electronic healthcare databases in real life practice settings.

The data were retrieved from the IRST electronic clinical data record archive and the following administrative region-wide databases made accessible online to us by the Department of Health of the Regional Administration:

(a) the hospital discharge database, from which we extracted information about any discharge of residents of the Emilia-Romagna Region from any public and accredited private hospital in Italy, including the start date and the end date of hospitalization, the principal diagnosis, the procedures and services provided (coded according to the ICD-O 10), and their cost;
(b) the outpatient specialist assistance database, containing individual information of all outpatient specialist visits, clinical tests and procedures (coded according to a local extension of the International Classification of Diseases), and their cost in € according to the Emilia-Romagna regional tariffs [28];
(c) the hospice admission database, containing the main information about any single hospice admission;
(d) the home care database, containing the main information about the utilization (visits and days) of home care services;
(e) the emergency database, containing information about any single emergency admission, including date, procedures, diagnosis and cost;
(f) the drugs databases, containing information on all drug expenditures (aggregate information about the drugs used in different settings) and high-cost drugs administration (specifications of any single high-cost drug administration). Drugs are coded according to the Anatomical Therapeutic Chemical Classification System [29].

In the study area, cancer hospices are exclusively hospice centers supervised by hospital clinicians from multidisciplinary cancer care teams, where people who have stopped treatment to cure their disease receive supportive care on an inpatient basis. Home care, too, is under the responsibility of multidisciplinary teams. For these reasons, visits, tests and treatments prescribed in hospices and at home were included in the study. The hospice admission and home care databases do not contain the cost of the procedures and services delivered. The Regional Administration, however, provides annual estimates of cost of hospice care and home care services for the residents of each healthcare district and the percentage of each total that is attributed to cancer care. After calculating the costs of hospice and home care services attributable to cancer, we broke them down by disease group using the distribution of patients in the IRST electronic clinical data record archive by cancer site.

We had no access to information on cancer drug provision by territorial pharmacies, specialist visits and diagnostic tests not prescribed by hospital cancer specialists, or general practice services.

Total health expenditures for cancer care data in the study area were obtained from the Italian National Institute of Statistics [30]. Information on the resident population was obtained from the statistical service of the Regional Administration. Further specifications of data sources used for this study have been reported elsewhere [16].

The study was approved by the Ethics Committee at the Istituto Scientifico Romagnolo per lo Studio e la Cura dei Tumori (IRST) IRCCS of Meldola, Italy (ID: IRST100.37). The study was also conducted in accordance with the Italian standards for data protection and the 1964 Helsinki declaration and its later amendments. Data were collected and analyzed through a pseudoanonymized code. Patients gave their informed consent to access their data for governance and planning purposes. 

### 2.6. Data Analysis

After record linkage and before analysis, the ‘real world data’ collected for this project underwent a three-stage check. If needed, the linkage process was reviewed and the data sources were re-explored or re-analyzed.

The study endpoints included the following: (a) the annual number of cancer patients, the total healthcare cost, the cost per patient, the cost per capita (i.e., per resident), and the percent share of total health expenditure per capita, by disease group; (b) the percent distribution of the total annual disease-group-specific cancer care cost by cost item (i.e., surgical admissions, inpatient and day-hospital medical admissions, radiotherapy, drugs, outpatient care, emergency admissions, and home and hospice care); and (c) the percent distribution of the total annual cost of each cost item for cancer care by disease group.

Costs per patient and per capita were expressed in € and cents. Total costs (inpatient care cost + outpatient care cost + administered drugs cost) were expressed in €, with the amounts rounded to the nearest euro unit. Values in the Tables may not sum exactly to totals because of rounding.

## 3. Results

In 2016, 10,486 cancer patients recorded in the IRST electronic clinical data record archive received healthcare services relating to their disease. Their distribution by disease group is shown in the left-hand column of Table 1.

Table 1 also shows that the total cancer care cost incurred for this population was €81,170,121, with an average cost per patient/year of €7741 and an average cost per capita of a little less than €205. Blood cancer had the highest healthcare cost, amounting to over one-fourth of the total cost, followed by gastrointestinal cancer care. The cost per breast cancer patient was the lowest one. Although the management of thorax cancer had the highest cost per patient, the cost per capita and the total were relatively limited. Overall, the estimated cancer care cost represented 11.1% of the total local health expenditure.

Table 2 shows the percent distribution of the annual disease-group-specific cancer care cost by cost item. Drug delivery, representing nearly 28% of total cost, was relatively higher in the treatment of blood cancer. Surgical admissions, conversely, accounted for a larger share of total cost incurred in the treatment of gastrointestinal cancer and uro-gynecologic cancer. The share of the total cost accounted for radiotherapy was markedly greater among breast cancer patients. Conversely, surgical cost and home and hospice care cost incurred for this disease were lower than in the other disease groups.

Table 3 shows the percent distribution of the total annual cost for each cost item by disease group (the amounts in euros appear in the heading of the corresponding column). The treatment of blood cancer constituted almost one-third of the cost of medical admissions and nearly 40% of pharmaceutical spending and emergency admissions. Overall, the treatment of gastrointestinal cancer and uro-gynecologic cancer accounted for nearly 60% of the cost of cancer surgery, while the treatment of blood cancer and gastrointestinal cancer accounted for an equal portion of the cost of medical admissions. The cost of chemotherapy for blood cancer and breast cancer amounted to a little less than 60% of the pharmaceutical spending. A little less than 50% of radiotherapy expenditure was absorbed by breast cancer.

**Figure 2:**
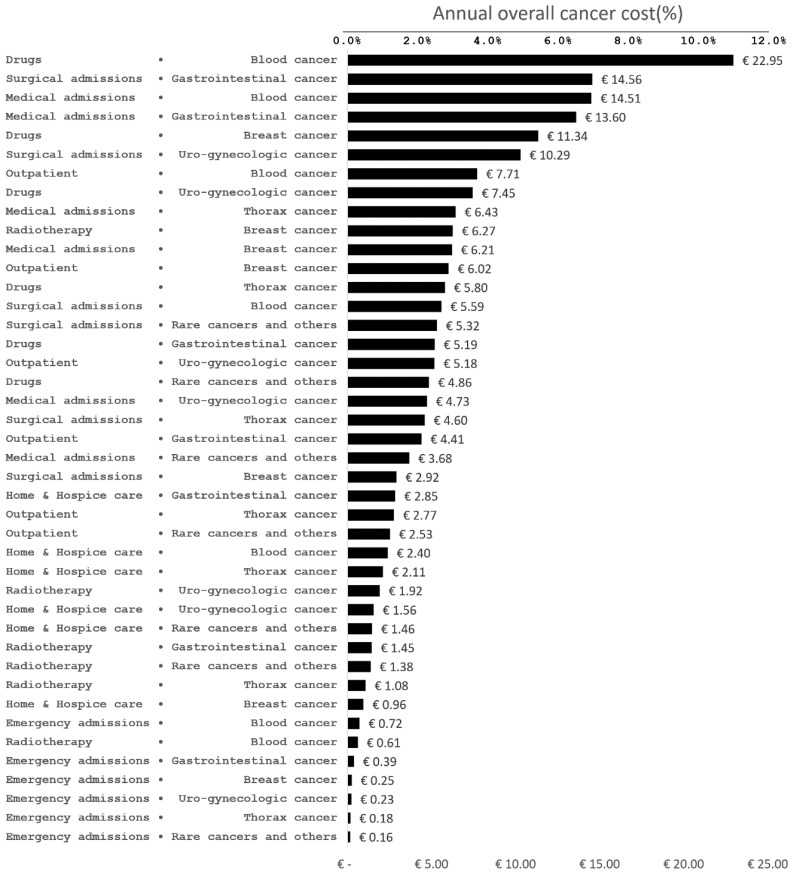
Annual cancer cost per capita in euros incurred for each cost item and each disease group, sorted in descending order. The horizontal bar represents the corresponding percentage of total annual cancer cost (use percent values in the upper horizontal axis as a reference) (Province of Forlì-Cesena, northern Italy, year 2016).

Values in Figure 2 are the annual cancer cost per capita incurred for each cost item and each disease group. The horizontal bar represents the corresponding percentage of the total annual cancer cost (use percent values in the upper horizontal axis as a reference). The five highest cost items included: drugs (11.2% of total cancer expenditure) and medical admissions (7.1%) for blood cancer, surgical (7.1%) and medical admissions (6.6%) for gastrointestinal cancer, and drugs for breast cancer (5.5%). Overall, they accounted for 37.6% of the total cancer expenditure.

## 4. Discussion

In Europe, two problems have yet to be solved before a multidisciplinary approach to cancer care and to the control of the related costs becomes a fully accomplished reality: first, the creation of care pathways is still incomplete and, second, multidisciplinary teams have only a partial control over costs. Improving control over costs implies a process of cost ascertainment and cost reporting. The objective of this pilot study was to develop an ETL process for acquiring cross-sectional information from multiple sources and to calculate the cancer care costs along the whole care pathways. The results provided an empirical cross-sectional snapshot of the current cost for cancer care. The main finding was that the cost per capita and the share of the total public and private health expenditure were higher than the Italian national estimates [31], i.e., €205 versus €111 and, respectively, 11.1% versus 5%. In part, this depends on epidemiologic factors. The Romagna area has one of the highest total cancer prevalence rates in Italy [23], which is due to a combination of high incidence, good patient survival, and growing longevity of the population. Survival rates are especially high for the most common types of cancer. Further increases have occurred, in the last two decades, for those types (particularly breast cancer and colorectal cancer) that have been targeted by mass screening, the level of provision of which varies considerably across Italy. However, the observed differences in costs relative to the national estimates are too large to be entirely explained by these factors. We are convinced that an important contributing cause is the higher sensitivity of our methods in measuring the actual cancer-related cost, because we considered each single procedure and service delivered to the patient along the entire care pathway from diagnosis to end-of-life care.

The comparability of our data with literature data is hampered by heterogeneity in disease grouping and costing methodology [32]. The following, however, can be safely stated. First, drugs were confirmed to be the single main component of total cancer cost [33]. This finding explains the observation that blood cancer care alone accounted for more than one-fourth of the total cancer expenditure. Recently, Burns et al. have noted the high cost incurred in the treatment of malignant blood disorders in the European Union [34]. Since 2012, many new drugs have received market authorization, and several are now priced well above the 2012 level [35]. It must be considered, however, that there are differences across countries and settings. In particular, some studies have placed more emphasis on the general increase in the expenditure for targeted drugs [36,37,38]. Again, epidemiologic factors play a role. In the Romagna area, the increasing cost of drugs interacts with the high incidence of leukemia and non-Hodgkin lymphoma and the improvements in the prognosis of hematologic malignancies. The rates of leukemia and non-Hodgkin lymphoma are significantly above the Italian national average rates, which is supposedly associated with the extensive use of agricultural pesticides in the past decades [39]. Improvements in patient survival, too, have an impact on costs because they increase the number of chemotherapy cycles.

Gastrointestinal cancer care was the second highest cost item. Its cost pattern, with resource use being concentrated in the early phase of the disease and in the terminal one, was a plausible finding [40]. This group of diseases ranked first in total cost of surgical and medical admissions, and of home and hospice care—the latter two being mainly accounted for by colorectal cancer patients and, respectively, by elderly patients with inoperable gastric cancer.

Regarding breast cancer care, it must be considered that resource utilization depends closely on the tumor stage [41], and that the local population is targeted by an efficient mammography screening program. This situation is mirrored by the cost structure. Firstly, early detection resulted in a preponderance of conservative surgery. This explains both the comparatively low cost of surgical admissions and the high cost of radiotherapy. Secondly, early detection led to improved survival and this, coupled with the high incidence of the disease, increased the prevalence of women with a history of breast cancer. In turn, this was associated with a relatively high cost of outpatient care (follow-up visits). Incidentally, the impact of radiotherapy on total cost was considerably lower in this study than in other studies [42]. A well-defined and generally accepted cost methodology for performing economic evaluation studies in radiotherapy, however, is lacking [32].

Many observations indicate that a multidisciplinary approach to cancer care provides the best clinical practice [43,44,45,46]. The benefits of multidisciplinarity may also include an overall improvement in the appropriateness of the use of resources. Multidisciplinary teams are responsible for the whole care pathway. If informed of the economic consequences of their clinical decisions, and particularly of areas of low-value use of healthcare resources, they can select the most appropriate treatment setting, reduce variability in the treatment process, reduce inappropriate medical prescriptions, avoid all unnecessary medical interventions, prevent service duplications, reduce hospitalizations, decrease the duration of stays, ensure a correct timing of care, and prioritize interventions based on a qualitative review of medical procedures, an example of which is described in our previous article [16]. Involving staff from multiple disciplines helps to unify the team in the common goals of efficiency and quality.

Improving the understanding of cost generation requires a widespread use of health information systems. We have the uncommon possibility to access multiple complementary data sources that cover all stages of cancer management. Combining information from these platforms enables us to build a coherent framework for cancer cost assessment. We have reviewed these sources in order to determine how they could be used to monitor all cost items and to generate cost indicators [10,47]. The potential savings generated by the information technology infrastructure are worth the investment for their purchase and maintenance.

This study is to a large extent free of the potential biases arising from the serious problem of interregional healthcare migration. The INHS is heavily decentralized at the regional level and, consequently, is highly variable. Cancer patient mobility, in particular, is driven by the technological endowment of the 20 regional healthcare systems [48]. The Emilia-Romagna Region, one of the richest and best performing administrative regions of Italy, is the second biggest “net exporter” of hospital treatments. Outflows of resident cancer patients are modest, except for some rare diseases.

Nonetheless, this study has limitations. First, the use of electronic administrative health data for a cost description study in the oncology setting has never been tested in Italy, although there have been experiences in the area of quality of care assessment [49]. The main advantages of this data source include its accessibility and real-time availability. The disadvantages are that administrative data may be affected by inaccuracies and missing value problems and may lack certain clinical and demographic details. In particular, they cannot always capture complex clinical circumstances that may justify non-standard clinical decisions [16]. Furthermore, administrative data are housed in disparate, nonintegrated datasets. This, however, has a positive effect on the completeness of the case series, because linking different administrative data sources increases the sensitivity of case finding, that is, the proportion of eligible subjects who are identified. Given the specific design of our project, we will place particular attention in future research on differences in the completeness of data recording (if any) between the six disease-specific cancer care workgroups.

Another limitation of the study is that we were unable to evaluate cancer drug provision by territorial pharmacies, visits and tests not prescribed by hospital cancer specialists, or general practice services. Aside from data accessibility limitations, these health services are virtually impossible to relate to a specific cancer diagnosis using current data. The resulting cost underestimation, however, is probably very limited, since cancer care in Italy is traditionally centralized in hospitals.

One additional problem is that the comparability of our results with other settings is limited by differences between tariffs. We used regional tariffs to attribute a quota in € to each cost component, assuming that different providers have the same efficiency.

Finally, the study does not provide a conclusive, in-depth analysis of cancer cost. As a part a multi-scope research project, it was primarily intended to be a feasibility study. A more accurate estimate of costs along the whole care pathway for each disease group is another and separate objective of the project.

## 5. Conclusions

We conclude that the pilot experience described here shows that it is possible to extract from ordinary platforms sufficient information for an exhaustive estimate of all costs, and to make multidisciplinary teams more aware of, and more accountable for, their resource use. We believe that this can boost the creation and the diffusion of multidisciplinary care models, and improve the degree of appropriateness of local cancer care services.

## Figures and Tables

**Table 1:** Annual number of cancer patients, total healthcare cost, cost per patient, cost per capita, and percent share of total health expenditure per capita, by disease group (Province of Forlì-Cesena, northern Italy, year 2016).

Disease Group	Number of Patients	Total Cost		Cost per Patient (€)	Cost Per Capita (€)	Percent Share of Total Health Expenditure ^a^
		Amount (€)	Percent Distribution			
Blood cancer	2943	21,619,772	26.6	7346.17	54.50	2.9
Gastrointestinal cancer	1849	16,830,382	20.7	9102.27	42.43	2.3
Breast cancer	2759	13,482,037	16.6	4886.12	33.99	1.8
Uro-gynecologic cancer	1457	12,443,182	15.3	8539.29	31.37	1.7
Thorax cancer	600	9,105,226	11.2	15,165.62	22.95	1.2
Rare cancers and others	877	7,689,522	9.5	8771.26	19.38	1.0
All	10,486	81,170,121	100.0	7741.17	204.62	11.1

^a^ Total health expenditure data in the study area were obtained from the Italian National Statistical Bureau [30].

**Table 2:** Percent distribution of the total annual disease-group-specific cancer care cost by cost item (Province of Forlì-Cesena, northern Italy, year 2016) ^a^.

Disease Group	Item of Expenditure							Total
	Surgical Admissions	Medical Admissions	Radiotherapy	Drugs	Outpatient Care	Emergency Admissions	Home and Hospice Care	
Blood cancer	10.3	26.7	1.1	42.2	14.2	1.3	4.3	100.0
Gastrointestinal cancer	34.4	32.1	3.4	12.2	10.4	0.9	6.6	100.0
Breast cancer	8.6	18.3	18.5	33.4	17.7	0.7	2.8	100.0
Uro-gynecologic cancer	32.8	15.1	6.1	23.8	16.5	0.7	4.9	100.0
Thorax cancer	20.1	28.1	4.7	25.3	12.1	0.8	9.0	100.0
Rare cancers and others	27.5	19.0	7.1	25.1	13.1	0.8	7.4	100.0
All	21.2	24.1	6.2	28.2	14.0	0.9	5.4	100.0

^a^ The amount of each total annual disease-group-specific cancer care cost is reported in Table 1.

**Table 3:** Percent distribution of the total annual cost of each cost item for cancer care by disease group (Province of Forlì-Cesena, northern Italy, year 2016).

Disease Group	Cost Item ^a^						
	Surgical Admissions (€17,185,783)	Medical Admissions (€19,523,707)	Radiotherapy (€5,046,641)	Drugs (€22,864,622)	Outpatient Care (€11,364,573)	Emergency Admissions (€764,019)	Home and Hospice Care (€4,420,776)
Blood cancer	12.9	29.5	4.8	39.9	26.9	37.5	21.1
Gastrointestinal cancer	33.6	27.7	11.4	9.0	15.4	20.1	25.1
Breast cancer	6.7	12.6	49.3	19.7	21.0	12.9	8.4
Uro-gynecologic cancer	23.8	9.6	15.1	12.9	18.1	11.9	13.8
Thorax cancer	10.6	13.1	8.5	10.1	9.7	9.1	18.6
Rare cancers and others	12.3	7.5	10.8	8.4	8.8	8.4	12.9
All	100.0	100.0	100.0	100.0	100.0	100.0	100.0

^a^ In parentheses, amount of total annual cost of each cost item.

## Data Availability

The datasets generated and/or analysed during the current study are available from the corresponding author on reasonable request.
